# Molecular detection and maternal transmission of a bacterial symbiont *Asaia* species in field-caught *Anopheles* mosquitoes from Cameroon

**DOI:** 10.1186/s13071-021-05044-2

**Published:** 2021-10-17

**Authors:** Claudine Grâce Tatsinkou Maffo, Maurice Marcel Sandeu, Amen Nakebang Fadel, Magellan Tchouakui, Daniel Nguiffo Nguete, Benjamin Menze, Michael O. Kusimo, Flobert Njiokou, Grant L. Hughes, Charles S. Wondji

**Affiliations:** 1 Department of Medical Entomology, Centre for Research in Infectious Diseases (CRID), P.O. BOX 13591, Yaoundé, Cameroon; 2 Department of Animal Biology and Physiology, Faculty of Science, University of Yaoundé 1, P.O. Box 812, Yaoundé, Cameroon; 3 Department of Vector Biology, Liverpool School of Tropical Medicine, Pembroke Place, Liverpool, UK; 4 Departments of Vector Biology and Tropical Disease Biology, Centre for Neglected Tropical Diseases, Liverpool School of Tropical Medicine, Liverpool, UK; 5 Department of Microbiology and Infectious Diseases, School of Veterinary Medicine and Sciences, University of Ngaoundéré, Po Box 454, Ngaoundere, Cameroon

**Keywords:** Malaria, *Anopheles*, *Asaia*, Genetic diversity, Maternal transmission, *Plasmodium* detection, Cameroon

## Abstract

**Background:**

Malaria control relies mainlyon insecticide-based tools. However, the effectiveness of these tools is threatened by widespread insecticide resistance in malaria vectors, highlighting the need for alternative control approaches. The endosymbiont *Asaia* has emerged as a promising candidate for paratransgenic control of malaria, but its biology and genetics still need to be further analyzed across Africa. Here, we investigated the prevalence of *Asaia* and its maternal transmission in the natural population of *Anopheles* mosquitoes in Cameroon.

**Methods:**

Indoor-resting adult mosquitoes belonging to four species (*An. coluzzii*, *An. arabiensis*, *An. funestus* and *An. gambiae*) were collected from eight localities across Cameroon from July 2016 to February 2020. PCR was performed on the *Asaia*-specific 16S ribosomal RNA gene, and samples positive by PCR for *Asaia* were confirmed by Sanger sequencing and phylogenetic analysis. The vertical transmission of *Asaia* was investigated by screening *F*_1_ mosquitoes belonging to *F*_0_ *Asaia*-positive females.

**Results:**

A total of 895 mosquitoes were screened. We found 43% (384) *Asaia* infection prevalence in four mosquito species. Phylogenetic analysis revealed that *Asaia* from Cameroon clustered together with the strains of *Asaia* isolated from other parts of the world. In addition, seven nucleotide sequence variants were found with low genetic diversity (*π* = 0.00241) and nucleotide sequence variant diversity (Hd = 0.481). *Asaia* was vertically transmitted with high frequency (range from 42.5 to 100%).

**Conclusions:**

This study provides field-based evidence of the presence of *Asaia* in *Anopheles* mosquitoes in Cameroon for exploitation as a symbiont in the control of malaria in sub-Saharan Africa.

**Graphical abstract:**

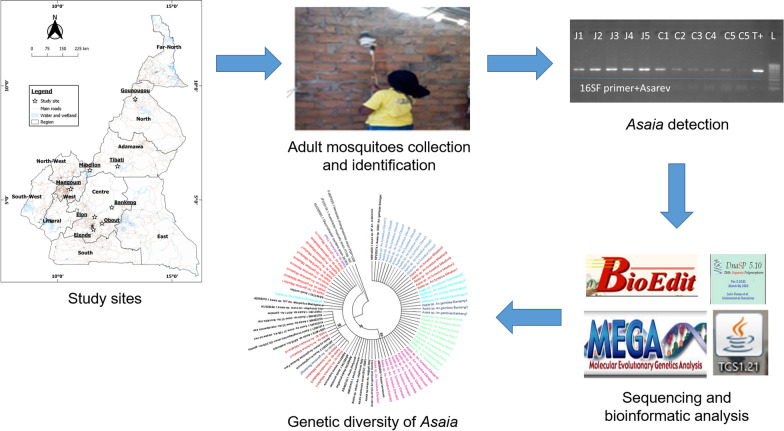

**Supplementary Information:**

The online version contains supplementary material available at 10.1186/s13071-021-05044-2.

## Background

Malaria is considered one of the most devastating diseases in sub-Saharan Africa. The World Health Organization (WHO) World malaria report 2019 recorded 216 million cases of malaria and 409,000 deaths worldwide in 2019 [1]. In Cameroon, malaria is endemic, with the whole population being at risk [2]. An estimated 23.6% of consultation in health centres, 68.7% of deaths in children under 5 years, and 16.9% of deaths in pregnant women are malaria-related cases [2, 3]. Malaria transmission in Cameroon is principally driven by species of the *Anopheles gambiae* complex (including *An. gambiae* sensu stricto [s.s.], *An. arabiensis* and *An. coluzzii*) and members of the *An. funestus* group (notably *An. funestus* s.s.) [4]. In this regard, insecticide-based vector control interventions, such as long-lasting insecticidal nets (LLINs) and indoor residual spraying (IRS) [5], have been the cornerstone of malaria prevention efforts.

However, widespread insecticide and drug resistance in mosquitoes and parasites, respectively, and the absence of an effective malaria vaccine are major obstacles weakening malaria control efforts across Africa, including Cameroon [6–8]. Therefore, the pursuit of novel, eco-friendly alternative tools is urgently needed to complement traditional malaria control strategies. In particular, the development of endosymbiont-based control strategies is receiving increasing attention, as microbes have desirable properties for vector control [9–11]. One approach, known as paratransgenesis, aims to inhibit the development of the parasites or to interfere with their competence and fitness by expressing effector molecules from symbiotic bacteria [12–14]. *Asaia*, a genus of acetic bacteria belonging to the *Acetobacteraceae* family [15, 16], has been identified as a promising candidate for paratransgenesis, since its characteristics support its use in vector control applications. *Asaia* has been isolated in malaria vectors such as *An. stephensi* [17], *An. gambiae* [18] and *An. funestus* [19]. It is localized in different organs of the *Anopheles* mosquito including the midgut, salivary glands and the reproductive system of both females and males [20]. *Asaia* is easy to cultivate and susceptible to genetic modification with exogenous DNA [18, 20]. It is a plant-mediated symbiont in which horizontal infection occurs through an oral route when mosquitoes feed on plant nectar [21, 22]. In addition to horizontal transmission, a venereal pattern during mating and vertical dissemination of *Asaia* to the next generation occurs via egg smearing [18, 23]. This highlights the symbiont’s ability to spread in mosquito populations [20]. For example, *Asaia* sp. SF2.1 has been demonstrated to be transmitted from female mosquitoes to their progeny through egg smearing [17]. *Asaia* has also been shown to facilitate the growth and reproduction of the mosquitoes seen during larval development in *An. stephensi* and *An. gambiae* [24, 25]. In addition, as shown in *An. stephensi*, co-localization of *Asaia* with a *Plasmodium* parasite in the host gut and the salivary gland plays a role in immune regulation through the activation of host antimicrobial peptides without self-inhibition [20].

Stable natural infection of *Asaia* in *Anopheles* mosquitoes has been shown in different species of *Anopheles* around the world [16, 17, 26] and more recently in African malaria vectors, notably *An. gambiae*, *An. funestus* and *An. coluzzii* [19, 27]. To our knowledge, only one report of *Asaia* has been documented in *An. coluzzii* and *An. gambiae* from Yaoundé, Cameroon, which focused on its detection by 454 pyrosequencing [28]. There is a paucity of available data on the prevalence of *Asaia* in other *Anopheles* species and in other localities across the country. In addition, the genetic diversity and the stability of the maternal transmission of *Asaia* in *Anopheles* mosquito are yet to be assessed in the Cameroonian context.

Investigating the genetic diversity and the maternal transmission of natural *Asaia* strains in *Anopheles* populations would allow a greater understanding of how this bacterium could influence malaria transmission in field populations and identify candidate strains for paratransgenesis. Here, we report on the detection, prevalence and maternal transmission of *Asaia* among field-caught *Anopheles* mosquitoes from different eco-geographical localities in Cameroon.

## Methods

### Study sites and sample collection

**Fig. 1:**
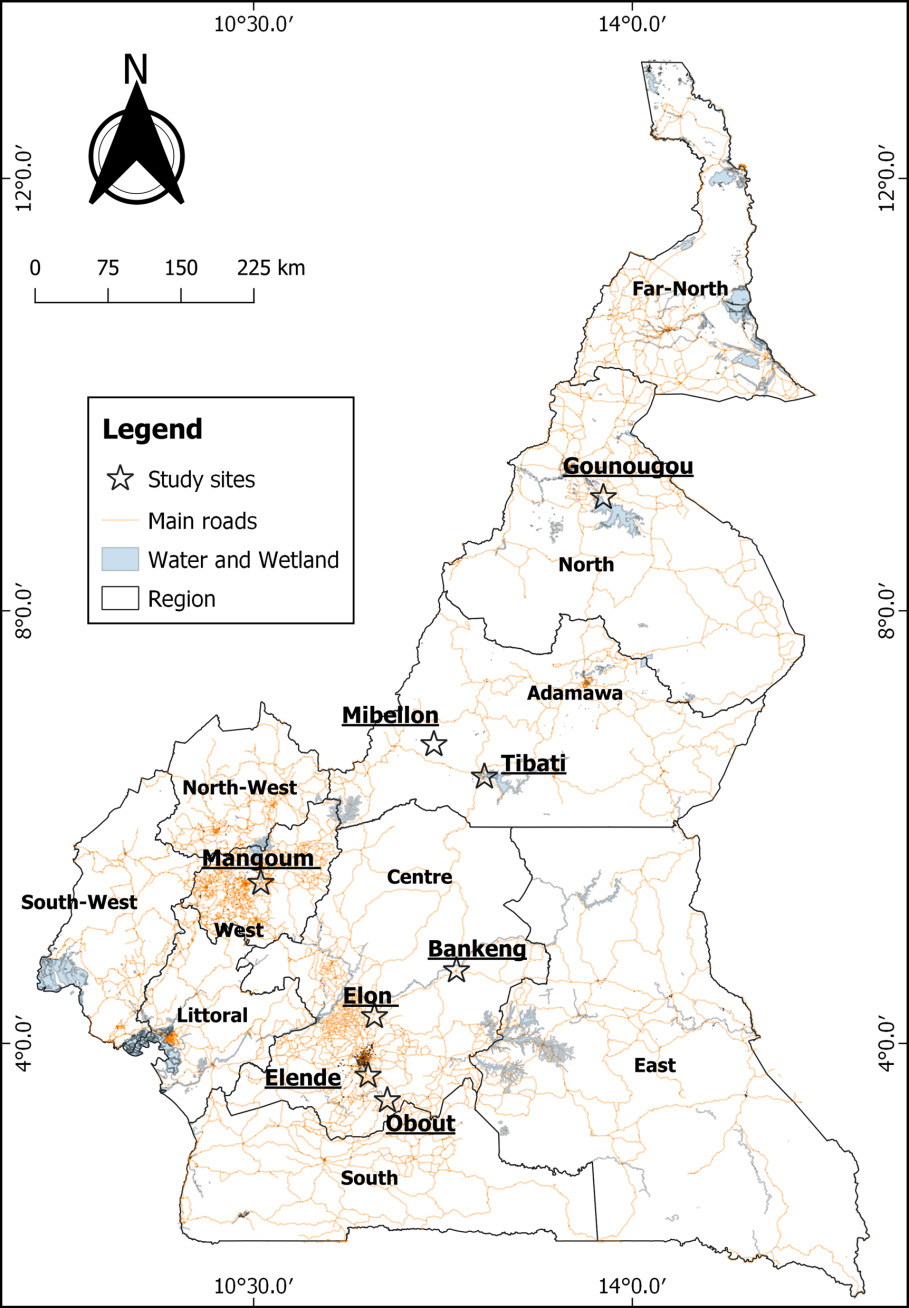
Map of the sampling sites. The study sites where the samples were collected are represented by stars. The map was constructed for this publication in QGIS 3.14 (https://www.qgis.org/fr/site/index.html) using country and regional boundaries from GADM (https://gadm.org/download_country_v3.html)

Mosquitoes were collected in eight localities in Cameroon, namely Gounougou (Northern Region 9°03′00″ N, 13°43′59″ E), Tibati (Adamaoua Region, 6°28′00″ N, 12°38′00″ E), Mibellon (Adamaoua Region, 6°46′ N, 11°70′ E), Mangoum (West Region, 5°28′60″ N, 10°34′0″ E), Bankeng (Centre Region, 4°40′26.4″ N, 12°22′30″ E), Elon (Centre Region, 4°15′0″ N, 11°37′0″ E), Elende (Centre Region, 3°41′57.27″ N, 11°33′28.46″ E) and Obout (Centre Region, 3°28′17.0″ N, 11°44′09.4″ E) (Fig. 1), from July 2016 to February 2020. The samples from Mibellon, Gounougou and Bankeng were collected in the rainy season, while those from Elende, Elon, Obout, Mangoum and Tibati were collected in the dry season. Indoor resting female mosquitoes were collected using electric aspirators between 06:00 a.m. and 09:00 a.m. following verbal consent from the chief and each household representative. The collected mosquitoes were then transported to the insectary at the Centre for Research in Infectious Diseases (CRID), Yaoundé, where they were morphologically identified following morphological identification keys for Afrotropical anopheline mosquitoes [29].

### DNA extraction and molecular species identification

Total genomic DNA (gDNA) of each adult mosquito was extracted using the Livak method [30]. Following extraction, the concentration and purity of the extracted gDNA were determined using a NanoDrop™ spectrophotometer (Thermo Scientific, Waltham, MA, USA) before storage at −20 °C. Short interspersed nuclear element (SINE)-based polymerase chain reaction (PCR) [31] and cocktail PCR [32] were performed to identify the different species of the *An. gambiae* complex and *An. funestus* group, respectively.

### *Plasmodium* infection rates

The detection of *Plasmodium* infection from each whole mosquito was performed using the TaqMan assay as described previously [33]. In this assay, two probes were used to check for the presence or absence of *Plasmodium* infection. The first probe, labeled with FAM, detects *P. falciparum*, and the second probe tagged with HEX detects *P. vivax*, *P. ovale* and/or *P. malariae.* This indicates that the effect of bacteria on *Plasmodium* development has been based only on the presence versus absence of *Plasmodium* infection.

### *Asaia* screening and sequencing

The presence of *Asaia* in the mosquitoes was detected with a diagnostic PCR using *Asaia-*specific primers 16SF (5′-TGG CGG ACG GGT GAG TAT C-3′) and Asarev (5′-AGC GTC AGT AAT GAG CCA GGT T-3′) [26] to amplify 676 base pairs (bp) of the 16S rRNA gene. The reaction mix was run for 5 min at 95 °C and cycled 35 times through 30 s at 94 °C, 30 s at 60 °C and 1 min at 72 °C. Finally, further extension was performed for 10 min at 72 °C. PCR products were then resolved and visualized on 1.5% agarose gel containing Midori green dye. If bands of the expected size were visible on the gels, the PCR products were cleaned up using Exonuclease I (Exo I) and Shrimp Alkaline Phosphatase (Exo-SAP protocol) according to the New England Biolabs protocol (NEB, Ipswich, MA, USA) and sent for sequencing. Sterile water was used as a negative control.

### Assessing the vertical transmission of *Asaia*

To assess the vertical transmission of *Asaia* in *Anopheles* mosquitoes, we investigated the presence of the bacteria in matching *F*_0_ female parent and *F*_1_ female *An. funestus* progeny from Elende. Blood-fed adult mosquitoes collected in Elende were transferred individually to paper cups and allowed to feed for 4–5 days on 10% sterile sugar-soaked cotton wool. Once fully gravid, females were allowed to oviposit individually by forced egg laying as described previously [34]. After hatching, larvae were reared to obtain *F*_1_ adults in isofamilies. *F*_0_ mosquitoes were then tested with their respective *F*_1_ for the presence of *Asaia*. Detection for the presence of *Asaia* by PCR in *F*_1_ mosquitoes was also performed, and the positive samples were sequenced to assess whether the *Asaia* strains from *F*_0_ to *F*_1_ individuals were the same. The *Asaia* sequences from *F*_1_ progeny were aligned and compared with the sequences of their respective *F*_0_ parents. This was done to confirm the vertical transmission pattern of *Asaia*.

### Phylogenetic analysis

Overall, 60 individual samples were analyzed using the 16S rRNA gene. The sample distribution included 10 *An. funestus* samples each from Mibellon, Obout, Elon and Elende, 10 *An. gambiae* samples from Mibellon, four *An. gambiae* samples from Bankeng, five *An. coluzzii* samples from Gounougou, and one *An. funestus* sample from Tibati. The number of samples utilized was based on the number of infected mosquitoes among the various species and the clarity of the sequences. Maximum likelihood phylogenetic trees were constructed using the *Asaia*-specific 16S rRNA gene target. The taxonomic relationships of the strains obtained from this study were inferred against GenBank sequence isolates. These existing isolates are presented in Additional file 1: Table S1. Additionally, these sequences were aligned with the ClustalW multiple sequence alignment tool in BioEdit software. *Neoasaia chiangmaiensis* (FJ887939.1), *Gluconobacter oxydans* (KU255083.1) and *Acetobacter tropicalis* (JF930138.1) were used as outgroups.

The evolutionary history was inferred by the maximum likelihood method using MEGA X based on the Jukes–Cantor model. The robustness of the individual branches was estimated by bootstrapping with 1000 replicates. All the sequences were deposited in GenBank under the accession numbers MW450601–MW450660. Genetic parameters including the number of nucleotide sequence variants, nucleotide sequence variant diversity (Hd), Tajima’s and Fu’s indexes and nucleotide diversity were computed using DnaSP 5.10.01. The TCS program was used to construct the haplotype network [35].

### Statistical analysis

Data were computed in MS Excel and analyzed using R software version 1.1.463. The Chi-square test was used to compare the prevalence of *Asaia* between different localities and species. The comparison of the prevalence of *P. falciparum* in *Asaia*-positive and *Asaia*-negative individuals was done using Fisher’s exact test.

## Results

### Prevalence of natural *Asaia* species and infection rates in *Anopheles* mosquitoes

Overall, 895 *Anopheles* mosquitoes belonging to four species, namely *An. funestus*, *An. gambiae*, *An*. *arabiensis* and *An. coluzzii*, were screened to determine the prevalence of *Asaia* sp. The results showed a general prevalence of *Asaia* sp. up to 43% (95% CI = 40–50%) (Table 1). All the species of *Anopheles* studied were found to be infected by *Asaia*, with infection rates of 43%, 48.4%, 33.7% and 20% for *An. gambiae*, *An. funestus*, *An. coluzzii* and *An. arabiensis*, respectively (Table 2).

**Table 1:** Infection rates of *Asaia* according to *Anopheles* species

Species	Tested	Infected	Infection rate (%)	95% Confidence interval
*An. coluzzii*	243	82	34	28–40
*An. arabiensis*	15	3	20	4–48
*An. funestus*	458	222	48.5	44–53
*An. gambiae*	179	77	43	36–51
Total	895	384	43	40–50

**Table 2:** Infection rate of *Asaia* in *Anopheles* mosquitoes according to species and locality

Localities	Species	Tested	Infected	Infection rate (%)	95% Confidence interval
Mibellon	*An. funestus*	122	63	51.6	42–61
	*An. gambiae*	94	53	56.4	46–67
Tibati	*An. funestus*	181	4	2.2	0.6–5
Obout	*An. funestus*	71	71	100	95–100
Mangoum	*An. gambiae*	48	0	0	0–7
Elon	*An. funestus*	41	41	100	91–100
Elende	*An. funestus*	43	43	100	92–100
Gounougou	*An. coluzzii*	242	81	33.5	28–40
	*An. arabiensis*	15	3	20	4–48
Bankeng	*An. gambiae*	37	24	64.9	47–80
	*An. coluzzii*	1	1	100	2.5–100

The prevalence of *Asaia* varied according to mosquitoes species*.* Indeed, *Asaia* prevalence was significantly higher in *An. funestus* than in *An. coluzzii* (*χ*^2^ = 3.39, *df* = 1, *P* = 0.00028) but was similar to the infection rate of *An. gambiae* (*χ*^2^ = 1.33, *df* = 1, *P* > 0.05) and *An. arabiensis* (*χ*^2^ = 3.64, *df* = 1, *P* = 0.142). Likewise, the prevalence of *Asaia* in *An. gambiae* did not differ from that in *An. coluzzii* (33.7%) (*χ*^2^ = 3.39, *df* = 1, *P* = 0.066) or *An. arabiensis* (*χ*^2^ = 2.15, *df* = 1, *P* = 0.414). Focusing on the different localities and *Anopheles* species, the infection rate of *Asaia* registered in *An. funestus* from Mibellon was 51.6%, compared to 2.2% from Tibati. The prevalence of *Asaia* was 0% in *An. gambiae* from Mangoum, whereas the prevalence was 100% in *An. funestus* from Obout, Elon and Elende. In *An. gambiae* populations, 56.4% and 64.9% were *Asaia*-positive in Mibellon and Bankeng, respectively. *Anopheles funestus* mosquitoes from Mibellon were found to be more frequently infected by *Asaia* than those from Tibati (*χ*^2^ = 100.54, *df* = 1, *P* < 0.001), while *An. funestus* mosquitoes from Obout were more frequently infected than *An. funestus* from Mibellon (*χ*^2^ = 47.2, *df* = 1, *P* = 0.001). On the other hand, the prevalence of *Asaia* in *An. gambiae* from Mibellon was comparable to that of *An. gambiae* from Bankeng (*χ*^2^ = 0.47, *df* = 1, *P* = 0.490).

According to mosquito collection seasons, out of the 458 *An. funestus* mosquitoes tested, 336 were collected in the dry season and 122 in the rainy season (Table 3). The prevalence of *Asaia* during the dry (51.6%) and rainy (47.3%) seasons was the same for *An. funestus* (*χ*^2^ = 0.67, *df* = 1, *P* = 0.413), showing that season does not have an impact. Regarding *An. gambiae*, 131 mosquitoes were collected during the rainy season, with an *Asaia* infection rate of 58.8%, while only 48 were collected during the dry season, with no infection observed (Table 3). This suggests that the rainy season has a positive effect on the prevalence of *Asaia* in *An. gambiae* (*χ*^2^ = 47.14, *df* = 1, *P* = 0.0001).

**Table 3:** Infection rates of *Asaia* according to season

Seasons	Species	Tested	Infected	Infection rate (%)	95% Confidence interval
Dry season	*An. funestus*	316	159	50	45–56
	*An. gambiae*	48	0	0	0–7
Rainy season	*An. funestus*	122	63	51.6	42–61
	*An. gambiae*	131	77	58.8	50–67
	*An. coluzzii*	243	82	33.7	28–40
	*An. arabiensis*	15	3	20	4–50
	Total	895	384	43	40–50

### Phylogenetic analysis and genetic diversity of *Asaia* sp.

**Fig. 2:**
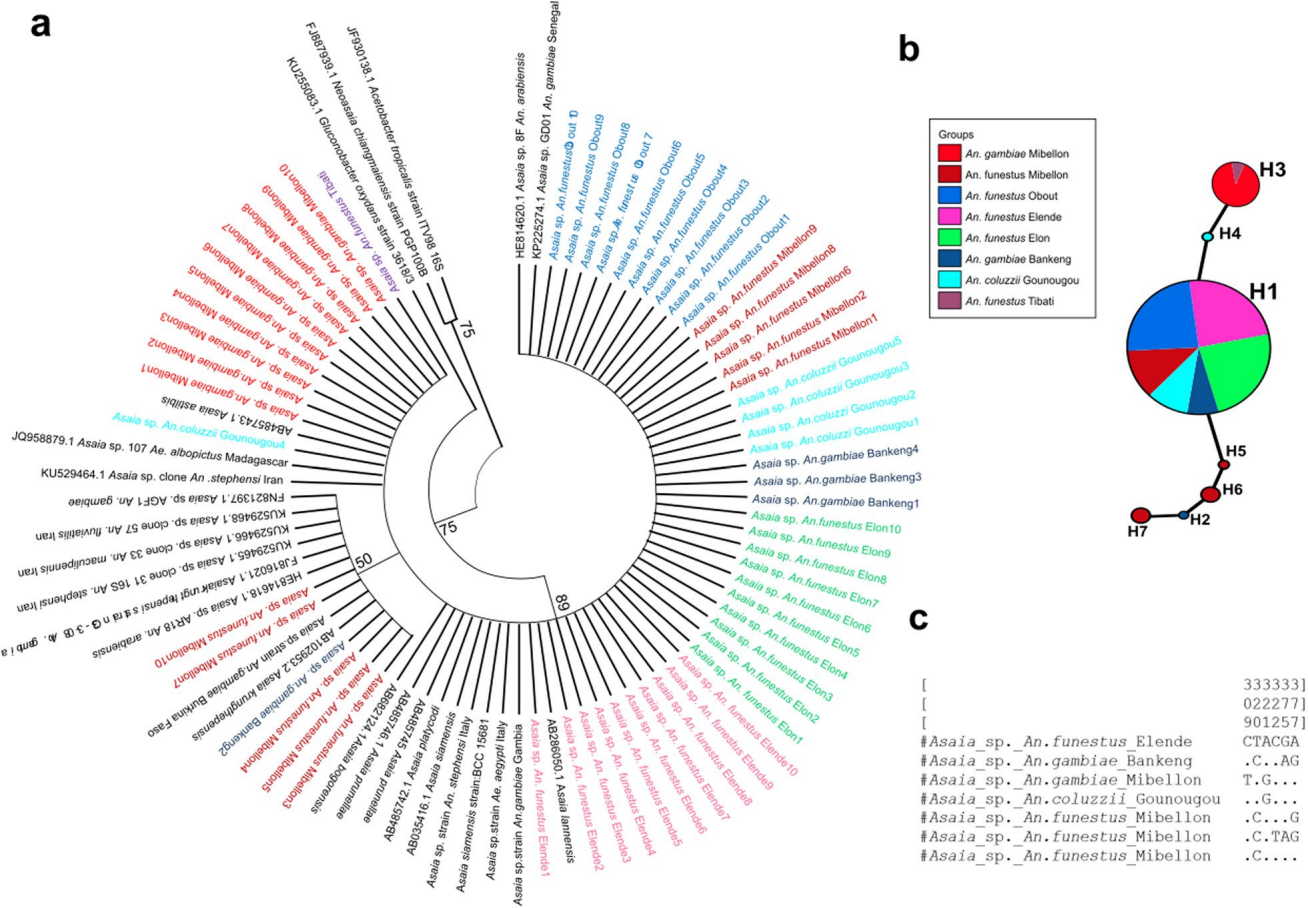
Genetic diversity of fragment of 16S rRNA gene for the detection of *Asaia* sp. in *An. coluzzii*, *An. gambiae* *and An. funestus* at Mibellon, Tibati, Gounougou, Obout, Elende, Elon and Bankeng. **a** Phylogenetic tree of the sequences based on the 16S rRNA gene. *Asaia* sequences of the *Anopheles* populations were used to construct the phylogenetic tree based on the maximum likelihood method with 1000 bootstrap replicates. The sequences of *Asaia* were aligned against close reference sequences. The bootstrap values under 50% were discarded. **b** Nucleotide sequence variant network. Nucleotide sequence variant networks were constructed using *Asaia* sequences in the TCS program. H1–H7 represent the different nucleotide sequence variants, and each color represents each mosquito species according to locality. **c** Alignment of nucleotide sequence variant showing the polymorphic sites

The 470-bp sequences obtained were subjected to BLAST search in GenBank (NCBI), and all had homology with *Asaia* sp. The relationship between *Asaia* sp. and *Anopheles* mosquitoes, notably *An. funestus*, *An. gambiae* and *An. coluzzii*, revealed a clustering of the same species of *Asaia* irrespective of the locality or species (Fig. 2a). Comparison of the sequences obtained to the reference revealed close relatedness of *Asaia* strains in five out of the 10 samples (50%) of *An. funestus* mosquitoes from Mibellon, and all the *An. funestus* (100%) from Elende, Elon and Obout. The strains shared close similarity with *Asaia* strains *A. platycodi*, *A. prunellae*, *A. siamensis* and *A. lannensis* isolated from *An. gambiae* from Senegal [21], *Asaia* strain AE isolated from *Ae. aegypti* from Italy [23] and other *Asaia* strains isolated from *An. stephensi* from Italy [17].

Similarly, five out of 10 (50%) *An. funestus* mosquitoes and one out of the four (25%) *An. gambiae* from Bankeng were found to be infected by a strain of *Asaia* sp. that clusters with *A. krungthepensis* and *Asaia* strain AGF0 of *An*. *gambiae* from Burkina Faso [18]*.* Moreover, *Asaia* sp. found in the 10 *An. gambiae* mosquitoes from Mibellon, one *An. coluzzii* (20%, 1/5) from Gounougou and *An. funestus* from Tibati cluster with *A. astilbis.*

Genetic diversity parameters of *Asaia* sp. revealed seven distinct nucleotide sequence variants (Fig. 2b), and low genetic diversity (*π* = 0.00241) and nucleotide sequence variant diversity (Hd = 0.481) (Table 4). The result also showed six variable polymorphic sites (Fig. 2c) with slight nucleotide differences. Depending on the mosquito species, we recorded nucleotide diversity of 0.00128, 0.00278 and 0.00084 for *An. funestus*, *An. gambiae* and *An. coluzzii*, respectively (Table 4). The major nucleotide sequence variant H1 (*n* = 42, frequency = 70%) was present in three species of mosquitoes originating from four locations (Mibellon, Elon, Elende, Bankeng and Gounougou). This suggests that H1 could be the original nucleotide sequence variant. H3 (*n* = 11, frequency = 18.3%) was found in *An. gambiae* from Mibellon and *An. funestus* from Tibati. The nucleotide sequence variant H2 (*n* = 1, frequency = 1.6%) was present in one individual *An. gambiae* sample from Bankeng, while nucleotide sequence variant H4 (*n* = 1, frequency = 1.6%) was detected in *An. coluzzii* from Gounougou. H5 (*n* = 1, frequency = 1.6%), H6 (*n* = 1, frequency = 1.6%) and H7 (*n* = 1, frequency = 1.6%) nucleotide sequence variants were present exclusively in *An. funestus* from Mibellon (Fig. 2b). The overall Tajima’s *D* statistic (*D* = −0.29815) and Fu’s *F* statistics (*F* = −1.335) used to determine the proximity to neutrality were negative and non-significant for the *Asaia* strains circulating in the *Anopheles* population from the different localities (Table 4).

**Table 4:** Genetic parameters

Locality	Species	*n*	*S*	*h*	Hd	*π*	Tajima’s *D*	Fu’s *F*s	FuLiD	FuLiF
Gounougou	*An. coluzzii*	5	1	2	0.400	0.00084	−0.81650 ns	0.090	−0.81650 ns	−0.77152 ns
Mibellon	*An. funestus*	10	4	4	0.7333	0.00377	1.04827	0.361	1.23914 ns	1.33492 ns
	*An. gambiae*	10	0	1	–	0.00000	–	0	0.00000	0.00000
Obout	*An. funestus*	10	0	1	–	0.00000	–	0	0.00000	0.00000
Elende	*An. funestus*	10	0	1	–	0.00000	–	0	0.00000	0.00000
Elon	*An. funestus*	10	0	1	–	0.00000	–	0	0.00000	0.00000
Bankeng	*An. gambiae*	4	3	2	0.5	0.00319	−0.75445	1.716	−0.75445	−0.67466
Pooled	*An. funestus*	40	4	4	0.235	0.00128	−0.85441	−0.555	1.02694	0.50379
	*An. gambiae*	14	5	3	0.473	0.00278	−0.58260	1.456	−0.95239	−0.88944
Total	–	60	6	7	0.481	0.00241	−0.29815 ns	−1.335	1.16049 ns	0.81346 ns

### *Asaia* sp. is vertically transmitted and displays stability of infection in *Anopheles* mosquitoes

**Fig. 3:**
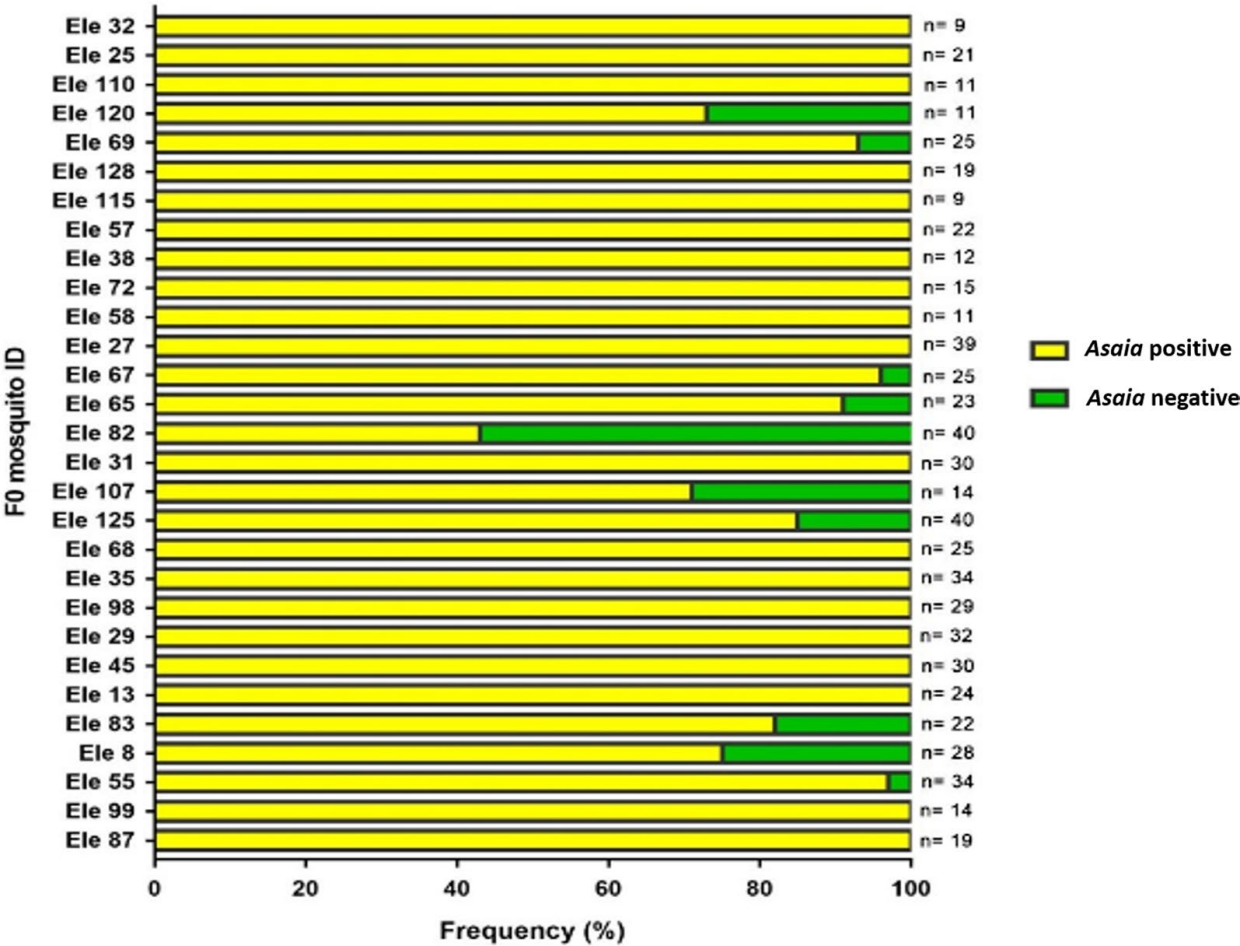
Transmission frequency of *Asaia* from *F*_0_ to *F*_1_. Ele *F*_0_ represents the Elende *F*_0_ female *Asaia*-positive sample, while Ele *F*_1_ is the Elende F_1_ mosquito. Frequency of transmission of *Asaia* in *F*_1_ progeny relative to *F*_0_-positive females. *n* represents the number of *F*_1_ mosquitoes tested for each *F*_0_

**Fig. 4:**
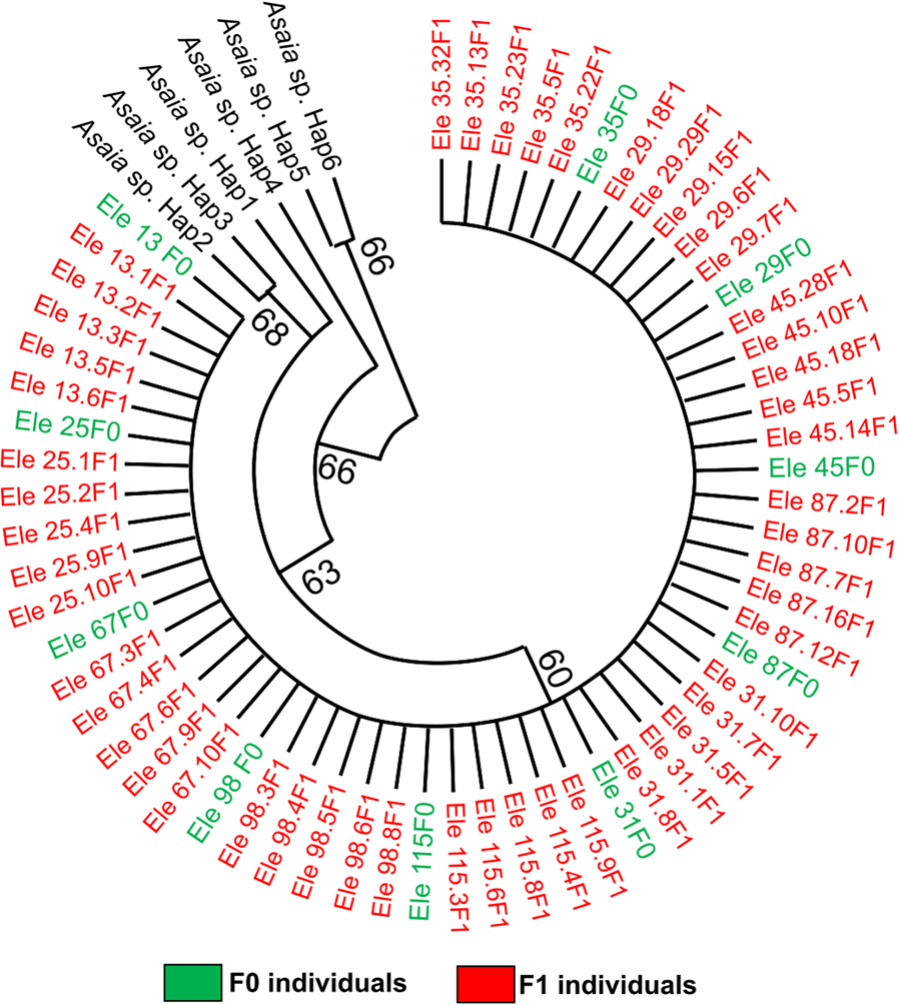
Phylogenetic tree showing the relationship between *Asaia* strains isolated in *F*_0_ and *F*_1_ mosquitoes. Ele *F*_0_ represents the *F*_0_ female *Asaia*-positive sample collected at Elende, and Ele *F*_1_ the respective progeny from Elende. Hap 1–6 represent the other haplotypes found in this study

Although *Asaia* is mainly maternally transmitted, horizontal transmission may occasionally occur in natural conditions. To confirm the vertical transmission in the infected mosquito species, we analyzed the infection status of 651 *An. funestus* *F*_1_ mosquitoes from Elende belonging to 29 isofamilies. We found that 601 *F*_1_ mosquitoes were *Asaia*-positive, with average vertical transmission frequency of 91.5% (range: 42.5–100%) (Fig. 3). In addition to the high transmission of *Asaia* in *F*_1_ mosquitoes, comparative phylogenetic analysis of the *Asaia* sequences from *F*_0_ to their *F*_1_ progeny revealed identical clustering (Fig. 4). Moreover, this prevalence of transmission could include both vertical and horizontal transmission because the internal control of testing progeny of *Asaia*-negative parents in order to exclude horizontal transmission through laboratory rearing was lacking in this study.

### *Asaia* has no effect on *Plasmodium* infection in *An. funestus* from Mibellon

**Fig. 5:**
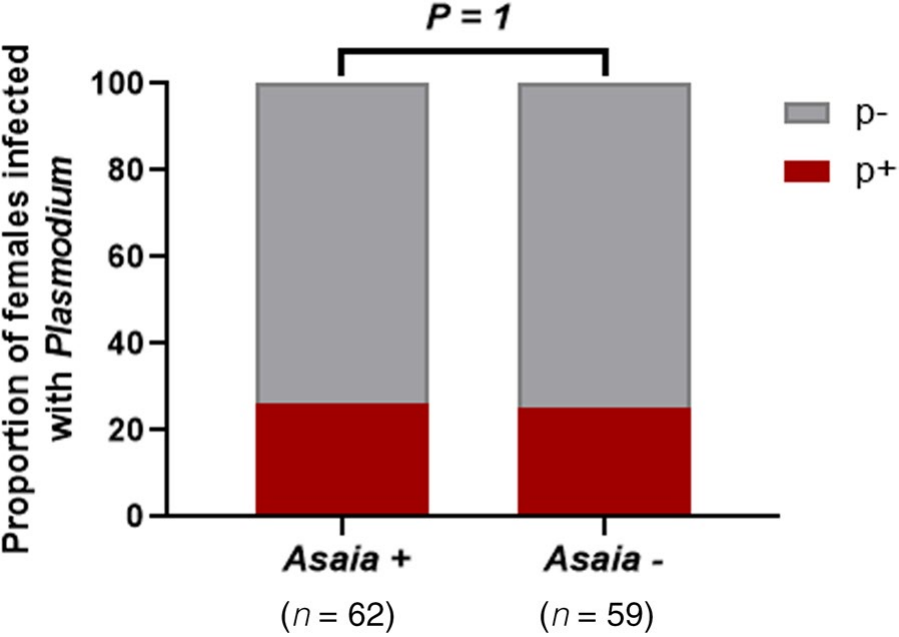
Correlation between *Asaia* and *Plasmodium* at Mibellon. The comparison of the prevalence of *Plasmodium* in *Asaia*-positive and *Asaia*-negative *An. funestus* mosquitoes at Mibellon was done using Fisher’s exact test. P+: *Plasmodium*-positive; P−: *Plasmodium*-negative

We compared the overall *P. falciparum* infection rates (based on the presence vs. absence of the *Plasmodium* infection) in *An. funestus* mosquitoes collected from Mibellon to determine a possible correlation with the presence of *Asaia* infection. Of the 122 mosquitoes sampled, only 25% had detectable *Asaia-Plasmodium* co-infections, as opposed to 51% *Asaia* mono-infections. Furthermore, only 31 out of the 122 (26%) mosquitoes were *Plasmodium*-positive. There was no association between the prevalence of *Plasmodium* in *Asaia*-negative and *Asaia*-positive groups (*χ*^2^ = 1.9, *df* = 1, *P* > 0.05) of *An. funestus* from Mibellon. Thus, *Asaia* has no effect on the natural *Plasmodium* infection status of *Anopheles* mosquitoes in Mibellon (Fig. 5).

## Discussion

This study provides an estimate of the prevalence of *Asaia* in natural populations of *Anopheles* mosquitoes across different localities of Cameroon. The mosquito species studied here have an overall *Asaia* prevalence of 43%. This prevalence is lower than the prevalence (70%) reported in *Anopheles* mosquitoes in Iran [26]. Likewise, it is also lower than the prevalence documented in the Centre Region of Cameroon, where *Asaia* frequency of 95% was observed in the midguts of *Anopheles* mosquitoes [28]. This difference could be explained by the seasonal, geographical and environmental variation in microbiota composition in *Anopheles* mosquitoes from the various study sites [36–38].

We also found that all the *Anopheles* species were infected by *Asaia*. This correlates with previous studies in the Centre Region of Cameroon where *Asaia* infection was recorded in *An. gambiae* and *An*. *coluzzii* mosquitoes [28]. This finding also parallels that of a study in Madagascar that demonstrated *Asaia* infection in *An. funestus* mosquitoes [27]. The presence of natural *Asaia* in several *Anopheles* species could be harnessed to develop a paratransgenic control method against *Anopheles* vectors responsible for malaria. The fact that *Asaia* has also been described in other species of mosquitoes such as *An. stephensi* [17] and *Ae. aegypti* [23] suggests that *Asaia* is not restricted to certain mosquito species. Rather, it can be widely distributed among many vector species. Moreover, *Asaia* has been reported to be stably associated with different mosquito species owing to its vertical and horizontal (through pre-adult and adult mosquito oral feeding) transmission potential. This also accounts for its dominance in the mosquito microbiome [17, 18, 23]. Furthermore, *Asaia* can be found in different niches, including mosquito breeding sites and also in flower nectars. These ecological segments constitute a suitable habitat for these bacteria [21].

The results obtained show variability in infection rates between localities in different species of *Anopheles*. This is explained by the heterogeneous routes in transmission including feeding on flowers, breeding site, and horizontal and vertical dissemination events [17, 21, 22]. The highest infection rates of *Asaia* were observed at Elon, Elende and Obout, which are all located in the forest region. This highest infection rate could be attributed to the abundance of plants and breeding sites. Indeed, it has been demonstrated that flower nectar is home to viable strains of *Asaia* [21].

The clustering of the *Asaia* strains obtained in this study with other known species of *Asaia* suggests that the strains of *Asaia* infecting *Anopheles* mosquitoes in Cameroon are not novel species. Further investigations are needed to identify each species of *Asaia* circulating in *Anopheles* populations across Cameroon. Moreover, the close relatedness with the *Asaia* strain isolated from other mosquito species from other countries suggests that introducing genetically modified *Asaia* strains in the *Anopheles* population will override genetic hurdles of mosquito populations isolated by reproductive barriers. These reproductive barriers often occur in endemic malaria settings and account for limitations observed with vector control strategies. In addition, the overall negative values for both neutrality tests (Tajima’s *D* and Fu’s *F*s tests) based on the 16S rRNA gene indicate an excess of the rare mutations in populations, which suggest a recent population expansion as already demonstrated in a previous study focusing on the genetic diversity of *Echinococcus granulosus* complex using mitochondrial DNA [39]. Also, analysis of the 16S rRNA gene confirmed that all the strains belong to the genus *Asaia*, but it was not possible to identify the species, and further analyses such as whole-genome sequencing could be used to better characterize *Asaia* strains of *Anopheles* mosquitoes in Cameroon.

This study demonstrated that *Asaia*-infected females transmit the infection with high frequency (91.5%) to their progeny, suggesting a vertical transmission of *Asaia*. Moreover, the close relatedness of the *Asaia* strains between *F*_1_ and *F*_0_ female mosquitoes suggests that *Asaia* displays stability of infection and reinforces the vertical transmission potential of the bacteria in *Anopheles* mosquitoes. This feature is crucial because it offers the possibility for introducing engineered *Asaia* into mosquito populations in the field which will spread over time and replace the wild-type population. The vertical transmission of *Asaia* has also been shown in other species of *Anopheles* [13, 17]. Our results suggest that *Asaia* is a promising candidate for bacterial engineering for the production of anti-plasmodium effectors. In fact, the high vertical transmission frequency of *Asaia* is not a surprising event, as it has already been demonstrated in *Anopheles* mosquitoes [17, 18]. Nevertheless, the high frequency of *Asaia* transmission recorded in this study could be due to a combination of horizontal and vertical dissemination events during co-feeding of *F*_1_ mosquitoes in the laboratory. Given that the presence of *Asaia* has not been tested in *Asaia*-negative parents, we cannot exclude the possibility that the DNA sequences detected in this study came from some sort of environmental contamination through sugar meals or other sources. Additional experiments would be of great interest to demonstrate actual infection, for example, showing maternal transmission of the bacteria from *Asaia*-negative samples and intracellular localization of the sequences. Also, different techniques such as fluorescence in situ hybridization (FISH) could be used to detect *Asaia* in different tissues (head and thorax, midgut and ovaries). Finally, the estimated rate of transfer of *Asaia* vertically from parents to progeny reported here also includes the rate at which *F*_1_ larvae acquired infection from laboratory sources.

Several distinct microbial-based approaches are currently being investigated [9–11]. However, it is unclear whether these approaches are compatible. For example, there is increasing evidence that microbe–microbe interactions influence colonization and abundance within the host [40–42], and these parameters are likely critical for successful control strategies. This is true for *Asaia* and *Wolbachia*, which appear antagonistic to one another [43, 44]. The recent finding of stable *Wolbachia* infection in *Anopheles* mosquitoes reinvigorates the use of this microbe for control of malaria [27, 45]. As such, there need to be careful considerations for the type of microbial control approach to be used in a particular region given that it may impede future control strategies.

*Asaia* has been shown to be a promising candidate for paratransgenic control of malaria. This is as a result of its negative effect on the development of *Plasmodium* at different stages [27]. Here we focused on the effect of *Asaia* on *Plasmodium* in natural populations. The data obtained show that in a field *Anopheles* population of Mibellon, the presence of *Asaia* did not affect the infection of mosquitoes by *Plasmodium*. These conclusions are in accord with the results of a recent study demonstrating that *Asaia* and *Wolbachia* do not influence *Plasmodium* infection in natural populations [27]. However, Bassene et al. showed that a strain of *Asaia* correlated negatively with *Plasmodium* infection in wild populations of *An. gambiae* sensu lato and *An. funestus* in Senegal [19]. Moreover, experimental infection studies need to be conducted to better evaluate the relationship between *Asaia* and *Plasmodium* infection in wild *Anopheles* mosquitoes. It will also be important to quantify the level of *Plasmodium* parasites and *Asaia* sp. in each *Anopheles* population to better determine this correlation.

## Conclusion

*Asaia* has been presented as a promising candidate for alternative control of malaria. Our study provides preliminary evidence of the circulation of *Asaia* in malaria vectors in Cameroon. The predominance of a nucleotide sequence variant in all the mosquito species suggests the feasibility of a paratransgenic control approach via the bioengineering of *Asaia* in malaria vectors. These data provide important initial baseline information towards developing potential strategies by exploring the possibility of utilizing this strategy for malaria control.

## Supplementary Information

**Additional file 1: Table S1.** References sequences and accession numbers.

## Data Availability

The data are part of a wider study of bacteria detection in *Anopheles* mosquitoes in Cameroon. Data are available from the corresponding author upon reasonable request.
