# Supplementary material for: Molecular detection and maternal transmission of a bacterial symbiont Asaia species in field-caught Anopheles mosquitoes from Cameroon
Source: Parasit Vectors. 2021 Oct 17;14:539. doi: 10.1186/s13071-021-05044-2 (PMC8522098; doi:10.1186/s13071-021-05044-2)
Supplement: Supplementary file 1 — Additional file 1: Table S1. References sequences and accession numbers. [file 13071_2021_5044_MOESM1_ESM.docx]

**Additional data**

| References | Accession numbers |
| --- | --- |
| *Asaia* *prunellae* | AB485745 |
| *Asaia* *bogorensis* | AB682124.1 |
| *Asaia prunellae* | AB485746.1 |
| *Asaia platycodi* | AB485742.1 |
| *Asaia* *siamensis* | AB035416.1 |
| *Asaia krungthepensis* | AB102953.2 |
| *Asaia astilbis* | AB485743.1 |
| *Asaia. lannensis* | AB286050.1 |
| *Asaia* sp. 8F | HE814620.1 |
| *Asaia* sp. AR18 | HE814618.1 |
| *Asaia* sp. GD01 | KP225274.1 |
| *Asaia krungthepensis* strain G3-3-08 | FJ816021.1 |
| *Asaia* sp. clone | KU529464.1 |
| *Asaia* sp. clone 31 16S | KU529465.1 |
| *Asaia* sp. clone 33 | KU529466.1 |
| *Asaia* sp. clone 57 | KU529468.1 |
| *Asaia* sp. AGF1 | FN821397.1 |
| *Asaia* sp. 107 | JQ958879.1 |
| *Neoasaia chiangmaiensis* | FJ887939.1 |
| *Gluconobacter oxydans* | KU255083.1 |
| *Acetobacter tropicalis* | JF930138.1 |
